# Managing an Acute and Chronic Periprosthetic Infection

**DOI:** 10.1155/2017/6732318

**Published:** 2017-11-14

**Authors:** Cristian Barrientos, Maximiliano Barahona, Rodrigo Olivares

**Affiliations:** Orthopaedic Department at Hospital Clinico Universidad de Chile, Santos Dumontt 999, Santiago, Chile

## Abstract

A case report of a 65-year-old female with a history of right total hip arthroplasty (THA) in 2007 and left THA in 2009 was presented. She consulted with our institution for the first time, on December 2013, for right hip pain and fistula on the THA incision. It was managed as a chronic infection, so a two-stage revision was performed. First-time intraoperative cultures were positive for *Staphylococcus aureus* (3/5) and *Proteus mirabilis* (2/5). Three weeks after the second half of the review, it evolved with acute fever and pain in relation to right hip. No antibiotics were used, arthrocentesis was performed, and a coagulase-negative staphylococci multisensible was isolated at the 5th day. Since the germ was different from the first revision, it was decided to perform a one-stage revision. One year after the first review, the patient has no local signs of infection and presents ESV and RPC in normal limits. The indication and management of periprosthetic infections are discussed.

## 1. Introduction

Periprosthetic infection is a complication that follows arthroplasty, whose incidence varies between 0.4 and 2% in the most recent studies [1]: 40% of infections occur within the first 2 years [2] and correspond to the main cause of primary early failure (<5 years) [3]. They are classified according to the time of evolution (Table 1). Among the risk factors (RFs) described are higher body mass index (BMI) at 30, diabetes mellitus (DM), use of corticoids, rheumatoid arthritis, tobacco use, cancer, MRSA colonization, chronic renal failure, and anemia. The risk increases directly in relation to the number of associated RFs [4–6].

## 2. Clinical History

A 65-year-old female ECF patient had a history of obesity and noninsulin-requiring diabetes mellitus, operated in 2007 for left total hip arthroplasty (THA) and in 2009 for right THA (both surgeries were performed in another center).

Her first visit to Clinical Hospital of Universidad de Chile, in December 2013, was for a 2-year history characterized by pain and functional impotence in the right hip, associated with recurrent febrile episodes and fistula in relation to scarring of the THA. In another center, it was managed with surgical lavage, debridement, and prolonged antibiotic treatments. General examinations, dated December 2013, include erythrocyte sedimentation rate (ESR, 54) and C-reactive protein (CRP, 30 mg/L).

Chronic periprosthetic infection was diagnosed (Tables 1 and 2). It was decided to suspend antibiotics (atb), and arthrocentesis under radiography was programmed after 3 weeks of the atb suspension. Positive polymicrobial culture was obtained from arthrocentesis for *Proteus mirabilis* and multisensitive *Staphylococcus aureus*.

**Figure 1:**
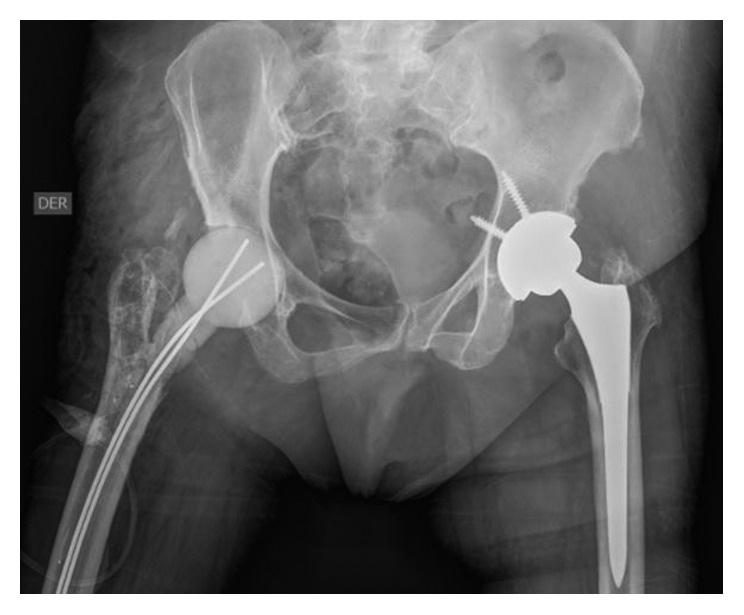
Postoperative radiograph after fistula resection, complete prosthesis removal, surgical lavage and debridement, tissue cultures, and vancomycin cement spacer were performed.

It was decided to perform an arthroplasty revision in two stages. First stage was scheduled for March 2014. Fistula resection, complete prosthesis removal, surgical lavage and debridement, tissue cultures, femoral intramedullary reaming, and vancomycin cement spacer were performed.  Figure 1 shows the postoperative radiograph. The cultures of intraoperative tissues obtained were positive for *Staphylococcus aureus* (3/5) and *Proteus mirabilis* (2/5), confirming the bacteriological diagnosis of arthrocentesis. After surgery, antibiotic treatment with intravenous vancomycin was restarted for 2 weeks, switching to oral ciprofloxacin for 40 days.

**Figure 2:**
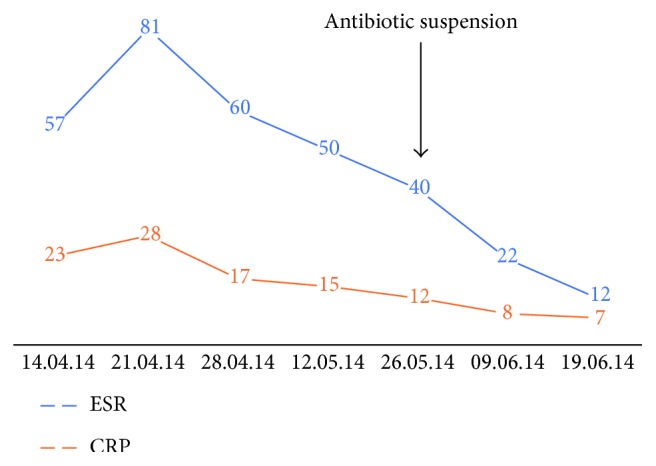
ESR and CRP since first visit to June 2014, just before second stage was performed.

It evolves favorably, without pain, without signs of systemic infection in the surgery wound.  Figure 2 shows the evolution of ESR and CRP, which were in decline, even after the atb suspension.

**Figure 3:**
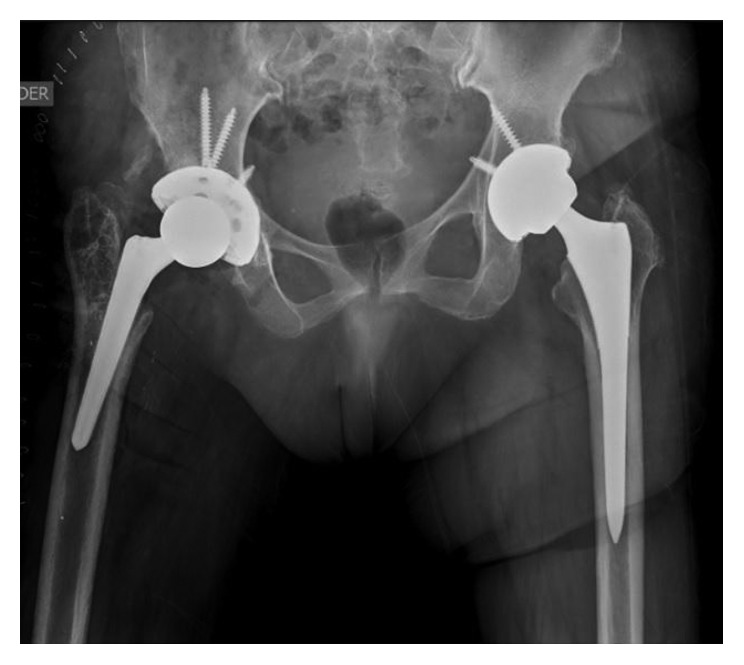
Postoperative radiograph after second stage and an uncemented total hip arthroplasty was performed.

Given the favorable evolution, it was decided to carry out the second stage. On July 2, 2014, spacer removal, taking of new cultures, and uncemented total hip arthroplasty were performed. Postoperative radiography is shown in Figure 3. After second stage, patient evolved favorably, managing to walk with 2 canes, declining HSV and CRP, and negative intraoperative cultures. It was discharged without antibiotic treatment.

She consulted one week after medical discharge, due to a one-day evolution episode, characterized by 39.5°C fever, right coxalgia, and secretion in relation to the surgery wound. Examinations are taken, in which leukocytosis 16,550, ESR 32, and CRP 22 are noticed. Hospitalization was decided at the same day of admission, and arthrocentesis under radiography was performed obtaining 145 cc of serohematic fluid that was sent for cultures. Hemocultures were negative at 5 days, and arthrocentesis cultures were positive to multisensitive negative coagulase *Staphylococcus*. Due to the time of evolution (Table 1) and the isolation of a microorganism different from the chronic infection, an acute periprosthetic infection was diagnosed. So, it was decided to perform a one-stage revision.

**Figure 4:**
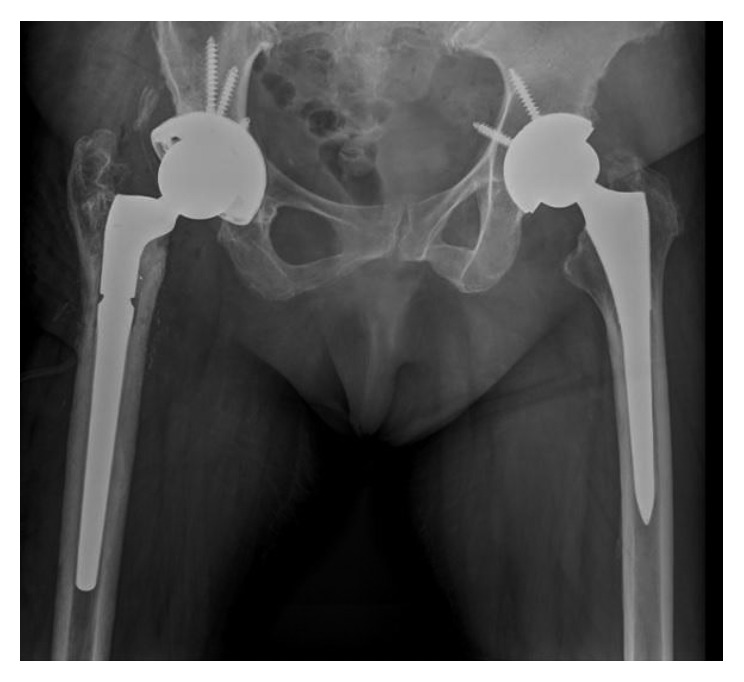
Radiograph after one-stage replacement for acute periprosthetic infection on July 2014.

On July 25, 2014, surgical lavage and debridement and prosthesis removal were done, 7 culture samples are taken, and total uncemented arthroplasty was performed. Postoperative radiography is shown in Figure 4. Five of seven intraoperative cultures were positive for multisensitive negative coagulase *Staphylococcus*, which is consistent with arthrocentesis. After three weeks of endovenous antibiotic treatment, in conjunction with infectology, it was decided to prescribe amoxicillin 875 mg + clavulanic acid 125 mg every 12 hr for 3 months at hospital discharge.

**Figure 5:**
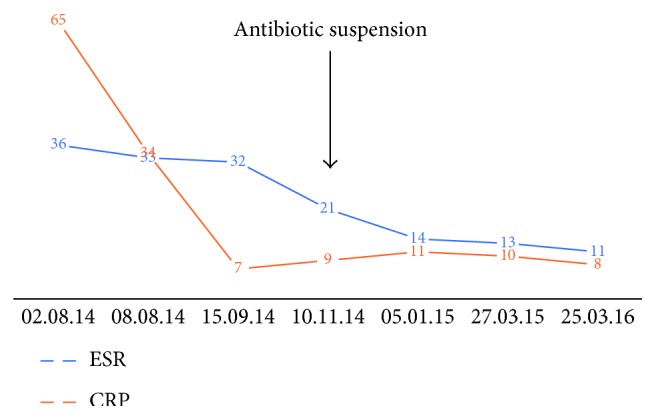
ESR and CRP after acute infection to March 2016, 20 months after last surgery.

It evolved favorably, with no new signs of systemic or local infection: movement without walking sticks and decrease of ESR and CRP, which was maintained after the suspension of the antibiotic (Figure 5).

**Figure 6:**
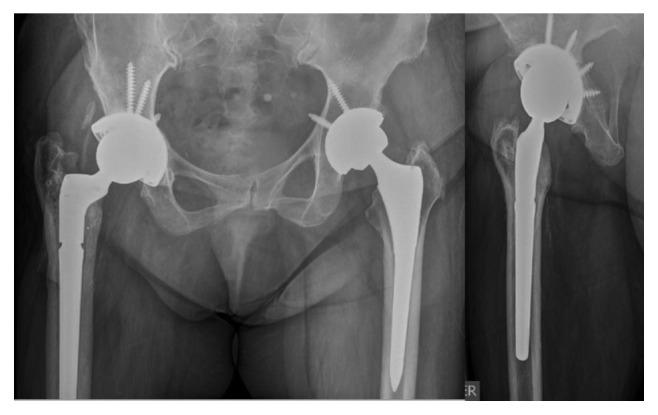
Radiograph at March 2017, 32 months after last surgery. No signs of loosening. Functional scores at final follow-up were HSS: 77.9 points, HOOS: 88.8%, and WOMAC: 93.8%.

The follow-up was until March 2017, that is, 32 months after the last surgery. Radiographs were taken (Figure 6), and functional scores were applied. The Harris Hip Score (HSS) [7] was developed to evaluate the results of hip surgery, the Hip Disability and Osteoarthritis Outcome Score (HOOS) [8] aimed to evaluate the patient's opinion about his hip and associated problems, and to this joint with or without osteoarthritis, the Western Ontario and McMaster Universities Osteoarthritis Index (WOMAC) [8] assessed the functionality and quality of life of patient with hip and knee pathology. The results obtained from the patient are as follows:

   HSS: 77.9 points (fair)
   HOOS: 88.8%
   WOMAC: 93.8%

## 3. Discussion

The clinical case described presents an important challenge given its chronic and then acute presentation. Therefore, a two-stage replacement was performed in the first instance and a one-stage replacement in the second instance.

Among the risk factors reported in the literature, the patient in the clinical case had 2 factors (obesity and DM) [9, 10]. The most frequently isolated bacteria in periprosthetic infection are *Staphylococcus aureus* (coagulase negative), Enterobacteria, and *Propionibacterium acnes* [1]. In physiopathology, it is important to know that the bacteria are organized by adhering to the prosthesis, multiplying and then invading neighboring tissues and the bloodstream. This corresponds to a continuous process, in which the bacteria are in a planktonic phenotype, that is to say, multiply rapidly, and in the phenotype of biofilm, in which, they synthesize adhesion proteins. Biofilm corresponds to a type of cellular organization, in which the bacteria form a true extracellular matrix which is composed of polysaccharides, glycoproteins, and nucleic acids. They also have a communication system called quorum sensing. This biofilm is a real barrier for antibiotics and also resists cleanings and surgical debridement [11].

For the diagnosis, MSIS has established the criteria described in Table 2 [2, 12, 13]. The patient in the case presented the 2 major criteria. The AAOS, in its guide to clinical practice in the diagnosis of periprosthetic infection, strongly recommends to always request HSV and PCR, since they have a high negative predictive value if both are normal and a high positive predictive value, close to 98%, if both are elevated [2]. In the case of total knee arthroplasty, if one of the two parameters is elevated, arthrocentesis should be done. In the case of THA, arthrocentesis is indicated, if both parameters are elevated and/or if there are compatible clinical and imaging findings [2, 12]. Recent studies demonstrate the utility of measuring levels of *α*-defensin and PCR in synovial fluid through ELISA test and to measure the presence of leucocyte esterase in synovial fluid with a urine test strip, all these measurements have high sensitivity for diagnosis [14–16].

The International Consensus on Periprosthetic Joint Infection emphasizes that if there is high clinical suspicion (anamnesis, physical examination, and radiology), studies should be performed to rule out periprosthetic infection, even though the criteria mentioned in Table 2 are not met [13]. Radiological findings may be radiolucency > 2 mm, accelerated component loosening, cement fractures, and subperiosteal reaction [2].

Another strong recommendation of AAOS is not to start antibiotics until getting cultures [12]. In addition, to decrease the percentage of false negatives, it is recommended to discontinue antibiotics 2 weeks prior to sampling [17]. In association with the above, it is also advisable to extend the culture for 2 weeks if the usual 5-day culture is negative, as this may improve the study's performance [12, 17]. In this case, this was done in both events. In the first instance, it was indicated to suspend and after 3 weeks to perform the puncture. In the case of July event, antibiotic treatment was not started until arthrocentesis was performed. This was useful in management, since it allowed the choice of antibiotic in both cases, and in particular in the second, to define that it was an acute infection and not a relapse, which clearly changed treatment planning [19].

Treatment alternatives included surgical cleaning and debridement (SCD), replacement at one stage (R1t), and replacement at two stages (R2t). These three associated with the use of antibiotics for a long time [12, 18].

SCD has a low success rate; in a systematic review performed by Romanò et al. [20], a success rate of 46% was investigated if 1 surgical grooming was performed (*n* = 170), while if 2 were performed, success rate rises to 52% (*n* = 175). Among the factors predicting SCD failure, the first SCD was performed after 48 hr of onset of symptoms, staphylococcal infection, BMI greater than 30, and immunosuppression. Therefore, its use should be restricted to acute unimicrobial and agent-detected infections other than *Staphylococcus*.

R1t would be indicated in those periprosthetic hip infections in which a microorganism is isolated, and the patient is in good general condition with maximum 2 comorbidities, immunocompetent and good bone stock. This was the treatment chosen for the July 2014 event, since a germ was isolated, more than 5 days had passed since the first symptoms and the patient was in good general condition [12, 21].

R2t corresponds to the gold standard for the management of periprosthetic infections, reporting a success rate between 80 and 100% according to the series. It is recognized as an aggressive and long-term procedure. In the planning, it is necessary to consider in the first time, the removal of all the materials of the prosthesis, without exception, of hybridity and hygiene with abundant saline solution, milling of the canal, taking of cultures, and leaving spacer with cement plus atb (according to agents isolated in previous arthrocentesis). Then, prior to the second time, the operative wound should be healthy and a follow-up of HSV and PCR should be maintained, which must be normal, after atb suspension. In addition, tissue cultures can be performed, which must be negative with less than 5 neutrophils per field prior to definitive arthroplasty [12].

In summary, the periprosthetic infection is of low incidence in the arthroplasty, but with great morbimortality. The most important thing is to take all measures to prevent it [5]. As for diagnosis, HSV, CRP, and arthrocentesis are essential elements. Finally, for periprosthetic infection the standard treatment is revision in two stages. R1t and SCD must be done only in selected cases like acute infection [21].

## Figures and Tables

**Table 1:** Classification of periprosthetic infection according to different authors [3, 4].

	Tsukuyama et al.	Toms et al.	Zimmerli et al.	Parvizi et al.
Acute	Less than 4 weeks	Less than 6 weeks	Less than 3 months	Less than 3 months
Subacute	N/A	N/A	>3 months	N/A
Chronic	>4 weeks	>6 weeks	>1 year	>3 months

**Table 2:** Diagnostic criteria for periprosthetic infections. For the diagnosis, 1 major criterion or 4 minor criteria are required [4, 13].

Major criteria	Acute: minor criteria	Chronic: minor criteria
Fistula (sinus tract)	CRP > 100 mg/L	CRP > 10 mg/L, ESR > 30

Two positive cultures for 1 microorganism(tissue or fluid)	Sinovial fluid WBC > 10,000 cells/*µ*L	Sinovial fluid WBC > 3000 cells/*µ*L
	Neutrophils > 90% on synovial fluid	Neutrophils > 80% on synovial fluid
	Purulence	Purulence
	1 positive culture (tissue or fluid)	1 positive culture (tissue or fluid)
	>5 neutrophils per high-power field in five high-power fields observed from histologic analysis of periprosthetic tissue at ×400 magnification	>5 neutrophils per high-power field in five high-power fields observed from histologic analysis of periprosthetic tissue at ×400 magnification
